# Antibacterial Effect of Fermented Pomegranate Peel Polyphenols on *Vibrio alginolyticus* and Its Mechanism

**DOI:** 10.3390/biology13110934

**Published:** 2024-11-16

**Authors:** Zhoulin Yu, Yucong Hong, Shuyan Zhao, Meng Zhou, Xiaohong Tan

**Affiliations:** 1 Innovative Institute of Animal Healthy Breeding, College of Animal Sciences and Technology, Zhongkai University of Agriculture and Engineering, Guangzhou 510225, China; yuzhoulin99@outlook.com; 2 Guangdong Provincial Key Laboratory of Aquatic Larvae Feed, Guangdong Yuequn Biotechnology Co., Ltd., Jieyang 515500, China; hongyucong13822047799@outlook.com (Y.H.); zhaoshuyan1990@outlook.com (S.Z.)

**Keywords:** *Vibrio alginolyticus*, fermented pomegranate peel polyphenols, antimicrobial activity, biofilm, oxidative stress

## Abstract

*Vibrio alginolyticus* frequently breaks out in aquatic animal breeding operations involving shrimp, and it can endanger human health through food and wound infections. The antibacterial effect and mechanism of fermented pomegranate peel polyphenols (FPPPs) on *V. alginolyticus* were investigated. The results indicated that FPPPs had a strong inhibitory effect on the growth of *V. alginolyticus*, and their minimum inhibitory concentration (MIC) and minimum bactericidal concentration (MBC) were 2 and 4 mg/mL. FPPPs significantly reduced biofilm formation and biofilm metabolic activity in *V. alginolyticus*, down-regulated the expression levels of *lafA*, *lafK*, *fliS* and *flaK* genes involved in flagellar synthesis and inhibited swimming and swarming motility (*p* < 0.05). Meanwhile, under the treatment of FPPPs, the activities of catalase (CAT) and superoxide dismutase (SOD) in *V. alginolyticus* were significantly reduced, and the levels of reactive oxygen species (ROS) and extracellular malondialdehyde (MDA) were significantly increased (*p* < 0.05). FPPPs also resulted in a significant increase in alkaline phosphatase (AKP) activity, protein and nucleic acid content, as well as conductivity from *V. alginolyticus* cultures. Scanning electron microscopy (SEM) images further revealed that *V. alginolyticus* treated with FPPPs showed leakage of intracellular substances, abnormal cell morphology and damage to cell walls and cell membranes, with the severity of the damage in a clear dose-dependent manner. Therefore, FPPPs can be used as a promising food-grade antibacterial agent, notably in seafood to control *V. alginolyticus*.

## Simple Summary

*Vibrio alginolyticus* is a Gram-negative bacterium that can infect aquatic animals such as fish, shrimp and shellfish, causing huge economic losses to aquaculture households and posing a potential threat to human health. The purpose of this study was to explore the antibacterial effect and mechanism of fermented pomegranate peel polyphenols as a new environmentally friendly antibacterial agent on *V. alginolyticus*. The results showed that fermented pomegranate peel polyphenols could exert antibacterial activity by inducing oxidative stress in *V. alginolyticus*, destroying the integrity of cell membrane and cell wall and inhibiting motility, biofilm formation and metabolic activity. This research will provide an important foundation for the application of fermented pomegranate peel polyphenols as a new and efficient antibacterial agent to control the infection of *V. alginolyticus* in live shrimp and other seafood.

## 1. Introduction

*Vibrio* is a genus of Gram-negative bacteria widely found in reservoirs, rivers, seas and other aquatic environments [1]. Most of these vibrios are non-pathogenic, and only a small percentage cause infectious diseases in aquatic animals or humans [2]. However, the outbreak and epidemic of vibriosis caused by this part of *Vibrio* not only cause harm to aquatic animals but also seriously affect human health [3,4]. *Vibrio alginolyticus* is a halophilic marine *Vibrio* in the genus *Vibrio*, and its number is among the highest in seawater vibrio [5]. *V. alginolyticus*, as a common opportunistic pathogen affecting and contaminating aquaculture aquatic products, is capable of causing massive mortality in marine fish, shrimp and shellfish [6,7,8]. In addition, humans can cause superficial wounds and eye and ear infections after contact with water contaminated with *V. alginolyticus* [9,10]. Meanwhile, human consumption of undercooked aquatic food carrying *V. alginolyticus* can even lead to diarrhea, gastroenteritis and septicemia [11,12,13]. Therefore, *V. alginolyticus* is a harmful bacterium that causes huge economic losses to aquaculture households and poses a potential threat to human health. 

Antibiotics can effectively prevent and control *Vibrio*, but long-term use will destroy the water environment and make pathogenic bacteria evolve drug resistance, and drug residues even endanger human health [14,15]. Hence, the development of environmentally friendly and safe new antibacterial agents has become an extremely urgent problem to be tackled in the field of aquaculture and the food industry. In recent years, medicinal plants and their extracts have received widespread attention for their favorable antimicrobial effects, such as cinnamaldehyde [16], shogaol [17], blueberry extract [18] and *Fructus Schisandrae Chinensis* extract [19]. Additionally, previous studies have shown that medicinal plants are rich in an assortment of active ingredients and nutrients [20,21,22]. They have the advantages of being natural, effective, inexpensive, non-toxic (or low toxicity) and less harmful to aquatic animals and the water environment, and it is not easy to generate drug resistance [23,24]. Therefore, it is reasonable and valuable to use them as antibacterial agents. 

Pomegranate (*Punica granatum* L.) is a fruit with high nutritional value, primarily distributed in subtropical and temperate regions [25]. In China, pomegranate has a long history of cultivation and rich planting resources and is a kind of plant which is available for medicine and food [26]. Pomegranate peel accounts for more than 40% the of pomegranate fruit and is typically discarded as a by-product of processing [27]. Nevertheless, pomegranate peel contains a variety of bioactive substances, among which polyphenols are the main phytochemicals [28,29]. Numerous studies have proved that pomegranate peel polyphenols (PPPs) can be used for lowering blood lipids and for anti-oxidation, anti-tumor, anti-inflammatory and antibacterial purposes [30,31,32,33]. These health benefits are attributed to the presence of polyphenol compounds such as punicalagin, ellagitannins, gallotannins and anthocyanins in PPPs [34,35]. Natural plant-derived foods are rich in polyphenols, but their bioavailability is low [36]. In the process of fermentation, beneficial microorganisms can secrete tannase, esterase, phenolic decarboxylase, glycosidase and other substances. At the same time, tannic acid, proanthocyanidins, gallic acid esters and other macromolecular-bound phenols were metabolized into quercetin, kaempferol, gallic acid and other free phenols to improve the biological activity and bioavailability of polyphenols [37]. The PPPs in this study were extracted by a factory process. Briefly, pomegranate peel is cleaned and crushed before fermentation, then extracted and concentrated with ethanol, and finally dried to obtain PPPs. Among them, fermentation has a similar effect with enzymatic hydrolysis, which can improve the efficiency of extracting polyphenols by destroying the cell wall of plants and promoting the release of bioactive components in pomegranate peel. Moreover, in our previous study, it was found that dietary supplementation with fermented pomegranate peel polyphenols (FPPPs) in feed can improve the survival rate of Pacific white shrimp (*Litopenaeus vannamei*) after infection with *V. alginolyticus* without negatively affecting their growth performance (unpublished article). This result is not only related to the fact that FPPPs can improve the antioxidant capacity and immunity of *Litopenaeus vannamei*, but it also may be related to the bacteriostatic effect of FPPPs on *V. alginolyticus*.

However, it is not known whether FPPPs have an antibacterial effect on *V. alginolyticus*, and there are few studies on the effect of PPPs on marine bacteria. Therefore, this experiment studied the antibacterial effect of FPPPs on *V. alginolyticus* and explored its possible antibacterial mechanism by measuring the growth curve, oxidative stress indexes, extracellular leakage content and bacterial morphology of *V. alginolyticus* treated with FPPPs. Furthermore, the effects of FPPPs on the biofilm formation, biofilm metabolic activity and motility of *V. alginolyticus* were also assessed. The results will provide a crucial foundation for the control of *V. alginolyticus* by FPPPs as a new environmentally friendly antibacterial agent in aquaculture and the food industry.

## 2. Materials and Methods

### 2.1. Bacterial Samples and Culture Conditions

*Vibrio alginolyticus* strain ZK2406 (GenBank accession number PP905135) was isolated and preserved from diseased *Litopenaeus vannamei* in a shrimp farm in Jiangmen City, Guangdong Province, China, by the Innovative Institute of Animal Healthy breeding, Zhongkai University of Agriculture and Engineering. Unless otherwise stated, *V. alginolyticus* was cultured in lysogeny broth (LB, Hope Bio-Technology Co., Ltd., Qingdao, China) liquid medium or LB solid agar plates containing 3% NaCl (*w*/*v*) at 30 °C throughout the study.

### 2.2. Reagents

The fermented pomegranate peel polyphenols (HPLC ≥ 60%) used in this study were purchased from Shaanxi Ciyuan Biotechnology Co., Ltd. (Xi’an, China). The main components of FPPPs were α-punicalagin (268.42 mg/g), β-punicalagin (274.27 mg/g), ellagic acid (18.78 mg/g), epicatechin (8.78 mg/g), catechin (3.31 mg/g) and gallic acid (1.34 mg/g). FPPPs was dissolved in dimethyl sulfoxide (DMSO, Sigma, St. Louis, MO, USA) and vortexed for 30 s at room temperature and then diluted with LB liquid medium to prepare an FPPPs stock solution with a concentration of 32 mg/mL that was stored at 4 °C. The final concentration of DMSO in the FPPPs stock solution was 0.1% (*v*/*v*), which had no apparent effect on the growth of *V. alginolyticus*. The FPPPs stock solution was diluted with LB liquid medium and the double dilution method to the appropriate concentration specified in each experiment. In addition, different concentrations of FPPPs used in this experiment had no significant effect on the pH of LB medium.

### 2.3. Determination of MIC and MBC

Minimum inhibitory concentrations (MICs) and minimum bactericidal concentrations (MBCs) of FPPPs against *V. alginolyticus* were detected using the standard broth microdilution method as partially modified by Yadav et al. and García-Herridia et al. [38,39]. The activated *V. alginolyticus* in LB liquid medium was diluted with phosphate-buffered saline (PBS) to a cell concentration of about 10^6^ CFU/mL. The 10^6^ CFU/mL bacterial solution was obtained by establishing the standard curve of bacterial colony-forming units-optical density at 600 absorbance value and diluting the bacterial liquid. Serial dilutions of the 32 mg/mL stock solution of FPPPs were carried out using LB liquid medium to obtain samples containing FPPPs at 4, 2, 1, 0.5, 0.25 and 0 mg/mL, respectively. In a sterile 96-well microtiter plate, 180 μL of LB liquid medium containing different concentrations of FPPPs was first added, and then 20 μL of *V. alginolyticus* solution with a concentration of 10^6^ CFU/mL was added. After incubation at 30 °C for 24 h, the optical density (OD) was measured at 600 nm using a Varioskan Lux Multimode microplate reader (Thermo Fisher Scientific, Dartford, UK). The lowest FPPPs concentration resulting in a lack of visible *V. alginolyticus* growth was considered the MIC. Liquid in the wells with a drug concentration greater than or equal to the minimum bacteriostatic concentration was selected to spread the plates, and the lowest drug concentration with less than 5 colonies on the plate was defined as the MBC.

### 2.4. Determination of Growth Curve

The growth curve was plotted to detect the killing kinetics of FPPPs against *V. alginolyticus* using the method of Liu et al. [40]. Briefly, *V. alginolyticus* suspensions (10^6^ CFU/mL) were inoculated at 1% volume into LB broth medium containing FPPPs at concentrations of 0, 1/8MIC, 1/4MIC, 1/2MIC and MIC, respectively. All samples were incubated at 30 °C for 200 rpm, and the cell density was continuously monitored at OD 600 nm for 24 h and the growth curve was drawn.

### 2.5. Determination of Biofilm Formation Ability

According to the method described by Qian et al. with modifications, the inhibitory effect of FPPPs on the biofilm formation ability of *V. alginolyticus* was determined by crystal violet staining [41]. The *V. alginolyticus* solution was diluted to the same OD600 value and 20 μL was pipetted onto a 96-well plate. Then, 180 μL LB broth medium containing FPPPs 0, 1/8MIC, 1/4MIC, 1/2MIC and MIC concentrations was added to the bacterial suspension and mixed evenly. After 48 h of static culture at 30 °C, the medium was discarded, washed with PBS and then incubated with 200 μL 1% crystal violet solution at room temperature for 20 min. After staining, the dye was aspirated and each well was rinsed 3 times with PBS and allowed to dry naturally. Finally, 200 μL of 33% glacial acetic acid was added to dissolve the biofilm, and OD was measured at the wavelength of 570 nm.

### 2.6. Determination of Biofilm Metabolic Activity

Determining the metabolic capacity of biofilms by measuring the metabolic activity of the cells in the biofilm has been a reasonable and widely research method [42]. The effect of FPPPs on the biofilm metabolic activity of *V. alginolyticus* was detected by a Cell Counting Kit-8 (CCK-8) assay [43]. The suspension of *V. alginolyticus* was inoculated into 96-well plate (200 μL/well) containing LB broth medium and incubated at 30 °C for 24 h; then the medium was discarded and the free bacteria were washed away with PBS. Then, 200 μL LB medium containing different concentrations of FPPPs was added. After 24 h of incubation at 30 °C, the medium was discarded and washed with PBS. Lastly, 180 μL PBS and 20 μL CCK-8 were added to each well, and OD was measured at 600 nm after incubation at 30 °C for 4 h protected from light.

### 2.7. Determination of Cell Wall Integrity

Alkaline phosphatase (AKP) usually exists in the periplasm, and the effect of FPPPs on the cell wall integrity of *V. alginolyticus* was evaluated by measuring the activity of extracellular AKP [44]. Bacterial suspensions (OD600 = 1.0) were added to LB broth medium containing FPPPs at concentrations of 0, 1/8MIC, 1/4MIC, 1/2MIC and MIC and then incubated at 30 °C for 8 h. In the end, 1 mL culture solution was centrifuged at 3500× *g* for 10 min, and the activity of AKP in the supernatant was detected by a commercial detection kit of Nanjing Jiancheng Bioengineering Insitute (Nanjing, China).

### 2.8. Determination of CAT, SOD and ROS

The intracellular catalase (CAT) and superoxide dismutase (SOD) activities and reactive oxygen species (ROS) levels of *V. alginolyticus* after treatment with different concentrations of FPPPs were determined according to the methods of Shivaprasad et al. and Li et al. [45,46]. The bacterial solution obtained after treatment with different concentrations of FPPPs for 8 h was centrifuged at 3500× *g* for 10 min. Bacterial precipitates were collected, washed and resuspended with PBS; then, bacteria were lysed with a SCIENTZ-IID ultrasonic cell crusher (SCIENTZ, Ningbo, China), and cellular debris was removed by centrifugation. The activity of CAT and SOD in the supernatant was determined by a CAT assay kit and SOD assay kit of Nanjing Jiancheng Bioengineering Insitute (Nanjing, China), respectively. Intracellular ROS levels were measured by Dichlorodihydrofluorescein diacetate (DCFH-DA) (Nanjing Jiancheng Bioengineering Insitute, Nanjing, China). The bacterial precipitates after FPPPs treatment were collected and washed with PBS and incubated in PBS containing DCFH-DA (10 μM) for 40 min. Eventually, 200 μL of the mixed solution was pipetted onto a 96-well plate and the fluorescence intensity was measured (excitation 488 nm, emission 525 nm).

### 2.9. Determination of MDA Content

Bacterial suspensions (OD600 = 1.0) were added to LB broth medium containing FPPPs at concentrations of 0, 1/8MIC, 1/4MIC, 1/2MIC and MIC, and then incubated at 30 °C for 8 h. In the end, 1 mL culture solution was centrifuged at 3500× *g* for 10 min, and the content of malondialdehyde (MDA) in the supernatant was determined by a commercial detection kit of Nanjing Jiancheng Bioengineering Insitute (Nanjing, China).

### 2.10. Determination of Nucleic Acid and Protein Leakage

The nucleic acid and protein of the biological macromolecules contained in the bacterial cell content have strong absorption of light at 260 and 280 nm, respectively [47]. Therefore, the content of extracellular nucleic acid and protein of *V. alginolyticus* can be estimated by measuring the absorbance at 260 and 280 nm. According to the steps described by Liu et al., the colorimetric method was used to determine the content of nucleic acid and protein released from *V. alginolyticus* under FPPPs treatment [48]. The *V. alginolyticus* suspension (OD600 = 1.0) was treated with FPPPs at 0, 1/8MIC, 1/4MIC, 1/2MIC and MIC for 8 h at 30 °C. After centrifugation at 3500× *g* for 10 min, the supernatant was obtained, and the content of extracellular nucleic acid and protein was determined at 260 nm and 280 nm. In addition, the total protein assay kit (Bicinchoninic Acid, BCA method) of Nanjing Jiancheng Bioengineering Insitute (Nanjing, China) was used to quantitatively determine the released protein content according to the instructions.

### 2.11. Determination of Conductivity

Studies have confirmed that when the cell membrane is injured, the electrolyte in the cell will flow out, resulting in changes in the conductivity of the extracellular culture medium. Conductivity was determined with reference to Lee et al [49]. Different concentrations of FPPPs were applied to the *V. alginolyticus* suspension (OD600 = 1.0) grown overnight in LB medium. After incubation at 30 °C for 8 h, the supernatant was obtained by centrifugation at 3500× *g* for 10 min. The conductivity of the supernatant was measured using a DDS-307A conductivity meter (INESA, Shanghai, China).

### 2.12. Motility

Swimming and swarming abilities play a crucial role for *Vibrio* in infecting host cells, and a loss of motility may affect bacterial adhesion and impair biofilm formation [50,51]. The effect of FPPPs on the swimming and swarming ability of *V. alginolyticus* was determined on solid LB plates containing 0.3% and 1.5% agar [52]. Different concentrations of FPPPs were added during the preparation of two solid media, so that the final concentrations of FPPPs were 0, 1/8MIC, 1/4MIC, 1/2MIC and MIC, respectively. Then, 5 μL of *V. alginolyticus* suspension (OD600 = 1.0) was aspirated in the center of the plate and incubated at 30 °C for 24 h. Subsequent photographs were taken using a gel imaging system and the diameters of the swimming circle and swarming circle were detected.

### 2.13. RNA Extraction and Gene Expression Analysis

*V. alginolyticus* was treated with different concentrations of FPPPs, and the bacteria were collected after 8 h of culture at 30 °C. The total RNA of *V. alginolyticus* was extracted using a Bacterial Total RNA Extraction Kit (Tiangen, Beijing, China). Then, the purity and concentration of each RNA were detected by a NanoDrop One microvolume-uv-spectrophotometer (Thermo Fisher Scientific, Waltham, MA, USA), and the integrity of total RNA was detected by 1% agarose gel electrophoresis. Finally, the total RNA (1000 ng) extracted in the previous step was reverse transcribed using an Evo M-MLV Mix Kit with DNA Clean for gPCR from Accurate Biotechnology (Hunan) Co., Ltd. (Changsha, China). The complementary DNA (cDNA) template of the product obtained by reverse transcription was stored in a refrigerator at −20 °C for subsequent fluorescence quantitative PCR (qPCR).

The primers were designed according to published articles and are listed in Table 1 [53]. Real-time quantitative PCR was performed using CFX Connect Real-Time PCR Detection Systems (Bio-Rad Laboratories, Hercules, CA, USA) with a Taq Pro Universal SYBR qPCR Master Mix Kit (Vazyme Biotech Co., Ltd., Nanjing, China) to detect the expression levels of lateral and polar flagellum synthesis regulation in *V. alginolyticus*. The qPCR reaction system and conditions were operated in accordance with the product instructions. Ultimately, the relative expression of each gene mRNA was analyzed by the 2^−ΔΔCt^ method with 16s rRNA (*16s*) as the internal reference.

### 2.14. SEM Observation

The effects of FPPPs on the cell morphology and cell membranes of *V. alginolyticus* were analyzed using scanning electron microscopy (SEM) [54]. *V. alginolyticus* was treated with different concentrations of FPPPs (0, 1/2MIC and MIC), and the organisms were collected by centrifugation at 3500× *g* for 10 min after incubation at 30 °C for 8 h. The organisms were washed using PBS and then resuspended in 2.5% glutaraldehyde solution and stored at 4 °C for 12 h. After centrifugation, the samples were dehydrated sequentially for 10 min with different dilutions of ethanol solutions (30, 50, 70, 80, 95 and 100%). Finally, the samples were vacuum placed on a sputtered gold-plated SEM scaffold and observed with a SU8100 scanning electron microscope (Hitachi, Tokyo, Japan).

### 2.15. Statistical Analysis

All experiments were conducted in at least triplicate. All the data were tested for normality and homogeneity of variance, followed by one-way analysis of variance and Duncan’s multiple range test. *p* < 0.05 suggested significant difference. The results are shown as means ± SD (*n* = 3), and SPSS 26.0 (IBM, New York, NY, USA) was used for statistical analysis.

## 3. Results

### 3.1. Inhibition of FPPPs on V. alginolyticus

**Figure 1:**
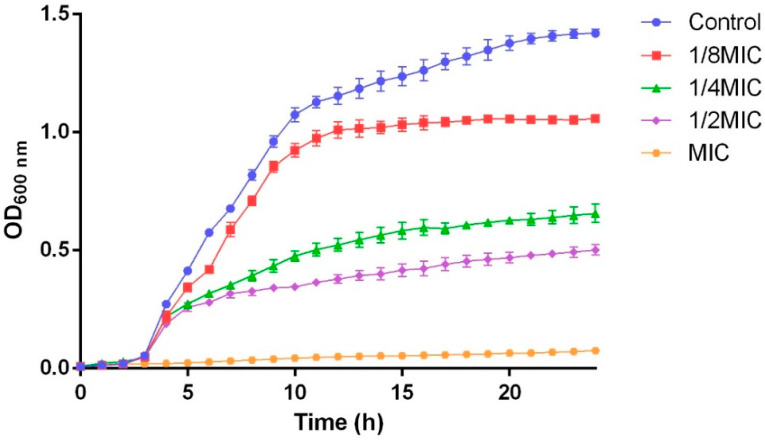
Growth curve of *V. alginolyticus* in LB under different concentrations of FPPPs. FPPPs concentration: control (0), 1/8MIC (0.25 mg/mL), 1/4MIC (0.5 mg/mL), 1/2MIC (1 mg/mL) and MIC (2 mg/mL). Error bars represent the mean ± SD of three replicates.

The MIC and MBC of FPPPs against *V. alginolyticus* were 2 and 4 mg/mL, respectively. Based on the MIC, five FPPPs concentrations were designed to explore the effects on the growth of *V. alginolyticus*, and the results are shown in Figure 1. On the whole, the growth rate of *V. alginolyticus* in the control group without FPPPs treatment was relatively fast; *V. alginolyticus* was in a growth retardation phase from 0 to 4 h, in a log phase from 4 to 10 h and in a stable growth phase after 10 h. FPPPs at 1/8MIC, 1/4MIC and 1/2MIC could partially inhibit the growth of *V. alginolyticus*, and FPPPs at the MIC could completely inhibit the growth of *V. alginolyticus*.

### 3.2. Inhibition of FPPPs on Biofilm Formation of V. alginolyticus

**Figure 2:**
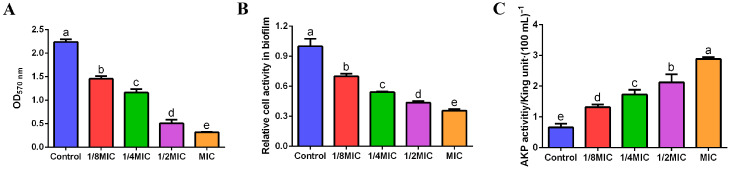
The effects of different concentrations of FPPPs on biofilm formation ability (**A**), biofilm metabolic activity (**B**) and extracellular AKP activity (**C**) of *V. alginolyticus*. Error bars represent the mean ± SD of three replicates. Different lowercase letters indicated significant differences (*p* < 0.05, Duncan’s test).

*V. alginolyticus* can form biofilms when cultured in 96-well plates, so the amount of biofilm formation can be detected by measuring the OD value of the decolorized solution after the elution of crystal violet. As shown in Figure 2A, the biofilm formation of *V. alginolyticus* in the control group was more, whereas the FPPPs-treated cells exhibited a significant dose-dependent inhibition of biofilm formation.

### 3.3. Inhibition of FPPPs on Biofilm Metabolic Activity of V. alginolyticus

CCK-8 can be reduced to a water-soluble orange-yellow formazan by dehydrogenases in bacterial cells, and its color depth is proportional to the metabolic activity of the cells. In this experiment (Figure 2B), the metabolic activity of *V. alginolyticus* biofilm in the group treated with FPPPs was significantly lower than that in control group (*p* < 0.05). With the increase of the concentration of FPPPs, the inhibitory effect on the metabolic activity of pathogenic bacteria biofilm was enhanced.

### 3.4. Effect of FPPPs on AKP Activity of V. alginolyticus

As shown in Figure 2C, the activity of the extracellular AKP of *V. alginolyticus* was increasing with increasing concentrations of FPPPs in the treatment group. As a result, FPPPs significantly enhanced the permeability of *V. alginolyticus* cell wall, which may be associated with its capability to disrupt the integrity of the cell wall.

### 3.5. Effect of FPPPs on Oxidative Stress Indexes of V. alginolyticus

**Figure 3:**
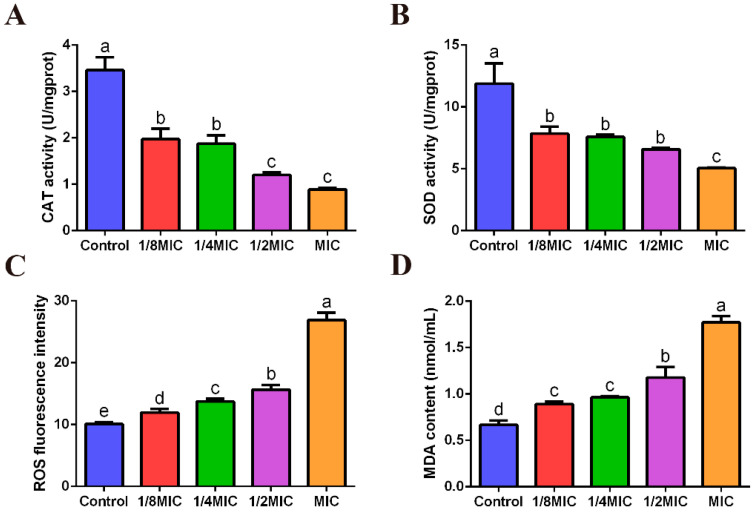
The effects of different concentrations of FPPPs on the intracellular CAT activity (**A**), intracellular SOD activity (**B**), intracellular ROS level (**C**) and extracellular MDA content (**D**) of *V. alginolyticus*. Error bars represent the mean ± SD of three replicates. Different lowercase letters indicate significant differences (*p* < 0.05, Duncan’s test).

The activities of intracellular CAT and SOD enzymes were significantly reduced and the level of ROS was significantly increased in *V. alginolyticus* treated with FPPPs in this experiment (Figure 3A–C). Moreover, the content of extracellular MDA of *V. alginolyticus* also increased with the increase in FPPPs concentration (Figure 3D). These above indices indicate that FPPPs can induce oxidative stress in *V. alginolyticus*.

### 3.6. Effects of FPPPs on Protein and Nucleic Acid Leakage of V. alginolyticus

**Figure 4:**
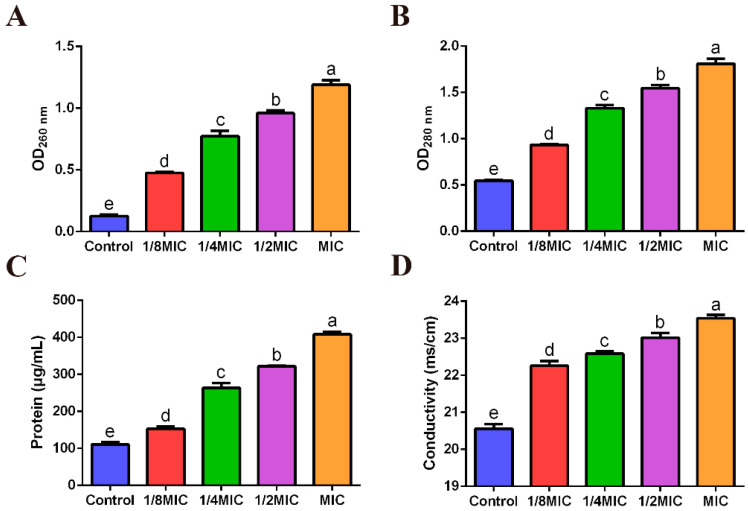
Effects of different concentrations of FPPPs on the extracellular DNA (OD260 nm) content (**A**) extracellular protein (OD280 nm) content (**B**), protein (detecting using BCA protein quantification kit) content (**C**) and extracellular conductivity (**D**) of *V. alginolyticus*. Error bars represent the mean ± SD of three replicates. Different lowercase letters indicate significant differences (*p* < 0.05, Duncan’s test).

As shown in Figure 4, the results of the determination of extracellular proteins using the protein assay kit (BCA method) were consistent with the absorbance method (Figure 4B,C). FPPPs at concentrations from 1/8MIC to MIC could significantly increase the content of extracellular protein and nucleic acid of *V. alginolyticus* (Figure 4A) (*p* < 0.05).

### 3.7. Effect of FPPPs on the Conductivity of V. alginolyticus Culture Medium

In the experiment, the conductivity of the extracellular medium of the control group without FPPPs was 20.56 ms/cm (Figure 4D). However, the conductivity of the extracellular medium of *V. alginolyticus* increased to 22.26, 22.58, 23.02 and 23.54 ms/cm after treatment with 1/8MIC to the MIC of FPPPs.

### 3.8. Inhibition of FPPPs on the Motility of V. alginolyticus

**Figure 5:**
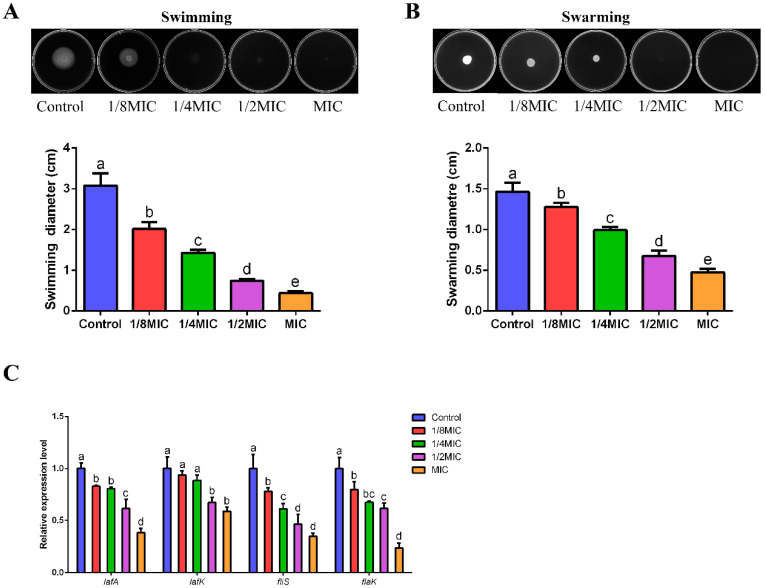
The effects of different concentrations of FPPPs on the swimming ability (**A**), swarming ability (**B**) and flagella formation related genes (**C**) of *V. alginolyticus*. Error bars represent the mean ± SD of three replicates. Different lowercase letters indicate significant differences (*p* < 0.05, Duncan’s test).

The results of motility showed that FPPPs dose-dependently reduced the swimming and swarming ability of *V. alginolyticus* compared with untreated group, while the MIC of FPPPs almost completely inhibited its motility (Figure 5A,B). Similarly, the 1/2MIC and MIC of FPPPs also significantly down-regulated the expression levels of *lafA* and *lafK* genes involved in lateral flagellar synthesis and *fliS* and *flaK* genes involved in polar flagellar synthesis (Figure 5C) (*p* < 0.05).

### 3.9. Effect of FPPPs on Cell Morphology of V. alginolyticus Under SEM

**Figure 6:**
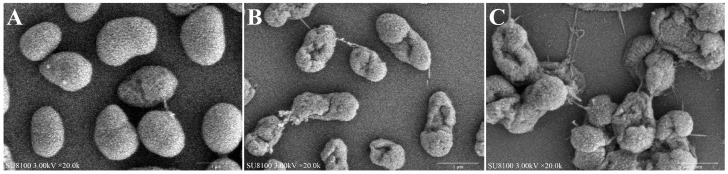
Effect of different concentrations ((**A**), (**B**) and (**C**): control, 1/2MIC and MIC, respectively) of FPPPs on cell morphology of *V. alginolyticus* under scanning electron microscope (SEM).

SEM images showed that the cell surface of *V. alginolyticus* treated without FPPPs was smooth, full in appearance and normal in shape (Figure 6A). The cell surface of the 1/2MIC FPPPs treatment group began to shrink and distort with some depressions, and there was slight adhesion between the cells (Figure 6B). The cell shape distortion of FPPPs treated with MIC was more serious, and the cell surface showed more obvious shrinkage and collapse (Figure 6C). Additionally, the leakage of intracellular substances as well as the imagery of cell aggregation and overlap were also observed.

## 4. Discussion

*V. alginolyticus*, as part of the normal microbiota of the aquatic environment, is not only recognized as a major pathogen in many outbreaks of *Vibrio* disease in marine organisms but is also an important pathogenic microorganism globally associated with seafood that can cause foodborne illness in humans [8]. In the natural environment, bacteria of the genus *Vibrio* can form a self-protective biofilm on the surface of seafood or during food transportation and processing, which not only makes it easier for the bacteria to withstand the stresses of the external environment but also provides an ideal platform for them to invade and infect other organisms more easily [55,56]. In addition, due to the characteristics of the bacterial biofilm, which leads to a high level of drug resistance, antibiotics that pollute the environment and easily cause side effects such as environmental drug residues are gradually replaced or banned [57,58]. Therefore, we have necessity to search for new safe antibacterial agents, which should not only have the effect of killing bacteria but also need to be able to pass through biofilm and not be blocked or neutralized by the biofilm.

Polyphenols have been widely reported to inhibit the growth of microorganisms, but there are relatively few studies on the bacteriostatic properties of PPPs, and only a few relevant papers have been published [59,60]. Belgacem et al. found that pomegranate peel extract has a good bacteriostatic effect on *Listeria monocytogenes* and fruits (apple, pear and melon), and they believe that this strong bacteriostatic ability may come from the polyphenols in pomegranate peel extract [61]. Coincidentally, Yemis et al. also confirmed that pomegranate peel extract contains polyphenols with antibacterial activity against *Cronobacter sakazakii*, so pomegranate peel can be used as a source of natural antibacterial agents [62]. Further, Chen et al. investigated the bacteriostatic effect of PPPs with a purity of 83.3% on the bacteriostatic effect of *Ralstonia solanacearum* [30]. They demonstrated that PPPs had a good antibacterial effect on *Ralstonia solanacearum*, which was even stronger than that of ellagic acid, catechin, gallic acid and other monomers in PPPs. Additionally, PPPs significantly inhibited the reproduction, motility and biofilm formation of *Ralstonia solanacearum*, even severely disrupting the morphological structure of the bacteria to the point of impairing the integrity of their cell membranes and cell walls. So far, the effect of FPPPs or PPPs on the antibacterial activity of *V. alginolyticus* and even pathogenic microorganisms in seawater and its mechanism have not been studied.

In this study, the bacteriostatic effect of FPPPs on *V. alginolyticus* was evaluated for the first time, and the results showed that FPPPs had a good bacteriostatic effect on *V. alginolyticus* with the MIC of 2 mg/mL. Meanwhile, the 1/8MIC to MIC of FPPPs enormously inhibited the growth of *V. alginolyticus*, and FPPPs at the MIC can even completely inhibit the growth of *V. alginolyticus*. Similarly, Liu et al. also confirmed that vanillic acid had good antibacterial activity against *V. alginolyticus* with the MIC of 1.0 mg/mL, and vanillic acid at the 1/2MIC and MIC had excellent inhibition on the reproduction of *V. alginolyticus* throughout the growth cycle [63]. In addition, plants or plant extracts such as *Fructus schisandrae*, *Rhizoma coptidis* and citral have been successively reported to have good bacteriostatic efficacy against *V. alginolyticus* [64,65,66]. However, since FPPPs are composed of a variety of monomers, we cannot be sure which monomer or monomers play the main antibacterial role. Fortunately, there has been a great deal of research demonstrating that monomers such as ellagic acid, catechins, gallic acid and chlorogenic acid have excellent antibacterial properties against all types of bacteria, especially punicalagin [67,68,69,70]. Unfortunately, due to the limitations of the experiment, we cannot discuss in detail the antibacterial ability of each chemical component in FPPPs in this study, but this may be a very meaningful research direction and the focus of our subsequent research in the future. In order to prevent the interference of different batches of commercial FPPPs on the experimental results, we purchased FPPPs twice at different time periods and determined their MIC against *V. alginolyticus*. The results of the two experiments showed that the MIC of FPPPs to *V. alginolyticus* was 2 mg/mL. Moreover, we learned from the merchants selling FPPPs that the polyphenol content was strictly determined according to the prescribed requirements when each batch of FPPPs was produced in the factory, and the production place of pomegranate is also consistent. Therefore, we believe that FPPPs between different batches will not affect the results and conclusions of this study. In addition, the effect of FPPPs as an antibacterial agent for food preservation on human health needs further study.

The flagellar-mediated motility of *Vibrio* is usually closely related to the formation of biofilm, bacterial colonization and virulence [71]. *V. alginolyticus* has two different types of flagellar systems, the unipolar flagellum involved in its swimming in the liquid environment and the lateral flagellum involved in its swarming on solid surfaces [72]. The FPPPs in this study down-regulated the expression levels of the flagellar synthesis-related genes *lafA*, *lafK*, *fliS* and *flaK* in *V. alginolyticus* while drastically inhibiting the swimming and swarming ability of *V. alginolyticus*. Moreover, the biofilm formation and biofilm metabolic activity of *V. alginolyticus* treated with FPPPs were significantly reduced. Previous studies have revealed that the motility of bacteria plays an essential part in the adhesion phase and biofilm formation on solid surfaces [73]. At the same time, antimicrobial-induced reductions in bacterial biofilm formation are often accompanied by cellular inactivation in the biofilm [74]. Coincidentally, Cao et al. confirmed that citral reduced the formation of *Vibrio parahaemolyticus* biofilms and inhibited the expression of the genes *lafA*, *flaA* and *flgM* involved in flagellar-mediated motility, as well as the swimming and swarming abilities [75]. Similarly, Liu et al. showed that vanillic acid inhibited the motility of *V. alginolyticus* by down-regulating the expression levels of the *lafA*, *lafK*, *fliS* and *flaK* genes while reducing its biofilm-forming ability [63]. Based on the above results and analysis, we speculated that FPPPs may inhibit the swimming and swarming ability of *V. alginolyticus* by down-regulating the expression levels of the *lafA*, *lafK*, *fliS* and *flaK* genes that mediate flagellar synthesis, thus affecting the biofilm formation ability and biofilm metabolic activity of *V. alginolyticus*.

Bacteria may suffer oxidative stress during survival, which can damage their normal cellular structure, and ROS and MDA are important indicators of oxidative stress [76]. Excessive ROS can cause injury to important biological molecules such as proteins, lipin and nucleic acids in cells, thus affecting the basic functions of cells and eventually leading to cell death [77]. MDA is the product of lipid peroxidation, and its massive accumulation may lead to the injury of cell membrane structure and function, resulting in the variation of its permeability [78]. On the contrary, antioxidant mechanisms are key to the bacterial response to oxidative stress, and a range of antioxidant enzymes such as CAT and SOD can mitigate oxidative damage by scavenging free radicals and thereby reducing oxidative damage [79]. We found that the activities of CAT and SOD in *V. alginolyticus* treated with FPPPs decreased obviously, while the ROS level and MDA content increased substantially. Coincidentally, Li et al. found that the intracellular ROS level and hydrogen peroxide concentration of *Vibrio mimicus* cultured with high-purity epigallocatechin gallate increased, and SOD activity decreased in a dose-dependent tendency [46]. Likewise, Lan et al. investigated the inhibitory mechanism of chitosan caffeic acid graft (CS-g-CA) against *Pseudomonas fluorescens*, and they concluded that CS-g-CA exacerbates the production of ROS and lipid peroxides in *Pseudomonas fluorescens*, which disrupts the respiratory and metabolic systems of the bacteria as well as the membrane integrity [47]. As a consequence, we believe that FPPPs can exert its antibacterial effect by inducing oxidative stress of *V. alginolyticus*.

The Majority of bacteria are protected from external environmental damage by a robust and elastic cell wall composed of peptidoglycan, but AKP will leak out of the cell after it is damaged [80]. The cell membrane is a natural barrier composed of phospholipids and various proteins to protect cells and participates in basic activities such as material exchange, cell metabolism and protein synthesis [81]. However, when the bacterial membrane is damaged, the permeability will increase, resulting in the leakage of intracellular proteins, nucleic acids, ions and respiratory chain disorder [82]. Now we found that the content of extracellular protein and nucleic acid and the conductivity of extracellular solution in *V. alginolyticus* increased after being cultured with FPPPs. This indicates that the intracellular proteins, nucleic acids and ions of *V. alginolyticus* have leaked, which may be related to the damage of bacterial cell membranes. In order to further verify our hypothesis, we also observed *V. alginolyticus* by SEM. Similarly, SEM images revealed that FPPPs severely damaged the cell wall and membrane of *V. alginolyticus* and produced cell content leakage, cell distortion, cell aggregation overlap and other phenomena. Similar conclusions were reached by Palamae, who demonstrated that a chitooligosaccharide–catechin conjugate disrupted *Vibrio parahaemolyticus* cell morphology as well as the integrity of the cell membrane and caused an increase in the conductivity and MDA content of cell cultures, as well as intracellular ion leakage [83]. Our findings were also in agreement with the previous SEM findings of *Vibrio parahaemolyticus* exposed to linalool and *Melissa officinalis* L. essential oil, which showed severe cell damage, disruption of the cell membrane and wall integrity, and leakage of intracellular substances [84,85]. Such results implies that FPPPs can inhibit the growth of *V. alginolyticus* through the mechanism of damaging cell membranes and cell walls as well as disrupting the normal morphology of cells. At the same time, it is precisely because FPPPs has the function of damaging the cell membrane of *V. alginolyticus*, which further aggravates the oxidative stress of *V. alginolyticus* and leads to the increase of MDA, a lipid peroxidation product of cell membrane. In summary, the antibacterial mode of FPPPs against *V. alginolyticus* is not only derived from a single mechanism of action but involves the cascade reaction of the entire bacterial cell.

## 5. Conclusions

This study demonstrated that FPPPs had a strong antibacterial effect on *V. alginolyticus*, and the MIC was 2 mg/mL. FPPPs can induce oxidative stress in *V. alginolyticus* and destroy the integrity of cell membrane and wall, leading to the leakage of intracellular substances such as proteins, nucleic acids and ions. Moreover, FPPPs can reduce the swimming ability and swarming ability of *V. alginolyticus* by down-regulating the expression of genes involved in flagellar synthesis and inhibit the formation and metabolic activity of biofilm. These findings provide an important foundation for the application of FPPPs as a new and efficient antibacterial agent to control the infection of *V. alginolyticus* in live shrimp and other seafood.

## Figures and Tables

**Table 1:** Primer sequences of genes used in real-time quantitative PCR.

Primer Name	Forward Primers (5′-3′)	Reverse Primers (5′-3′)	Source
*16s*	AAAGCACTTTCAGTCGTGAGGAA	TGCGCTTTACGCCCAGTAAT	[45]
*lafA*	CGCAGGTATCGGTGAAATCA	CCGAAGTCTGCACGAGAGCTA	[45]
*lafK*	GAATCGGGAACGGGTAAAGAA	GGTGAACGCGCCTTTTACAT	[45]
*fliS*	CTGGTGCGATTGAGCGCCTTATTCA	CGTCGATCAGCTGAGGCTCATTTTG	[45]
*flaK*	GTATCAAACACGGAAGCAAACG	TTCTAGGAGCTCAGGCGGTATT	[45]

## Data Availability

The datasets of the current study are available from the corresponding author upon reasonable request.
